# Prevalence of Anxiety, Depression, and Sleep Disturbances Associated With the COVID-19 Outbreak in Riyadh, Saudi Arabia

**DOI:** 10.7759/cureus.24838

**Published:** 2022-05-09

**Authors:** Rahaf F Alkahtani, Abdulrahman A Alomar, Abdulrahman F Alkanhal, Mohammad F Alhinti, Shahad E Alatoui, Rinad R Alrashidi, Ahmad Saleh

**Affiliations:** 1 College of Medicine, Imam Muhammad Ibn Saud Islamic University, Riyadh, SAU; 2 College of Medicine, University Of Hail, Hail, SAU; 3 Research Center, King Fahad Medical City, Riyadh, SAU

**Keywords:** depression, anxiety, insomnia scale, anxiety scale, insomnia, covid-19, pandemic

## Abstract

Background

COVID-19 became a global respiratory pandemic as it disrupted millions of lives and commerce. The implementation of strict lockdown measures to confine the outbreak can negatively affect people’s overall sleep quality and mental health. We aimed to assess the prevalence of sleep disturbance and the psychological impact associated with the spread of COVID-19 in Riyadh, Saudi Arabia.

Materials and methods

A cross-sectional study was conducted in Riyadh, Saudi Arabia. A nationally online questionnaire was sent to participants aged >18 years to assess their socio-demographic information, assessment of psychological status by Hospital Anxiety and Depression Scale (HADS), and assessment of sleep disturbance by Athens Insomnia Scale (AIS) and Insomnia Severity Index (ISI) scales.

Results

The total sample consisted of 399 participants. The mean age was 34.70 ± 12.57 years; predominant responses were from females (69.4%). The study sample was mostly made up of students (32.1%), and more than half of the participants (52.6%) were married. The prevalence of anxiety, depression, and insomnia was 38.6%, 33.1%, and 54.9%, respectively. Participants with comorbidities were significantly at higher risk of having symptoms of depression in comparison to subjects free from chronic diseases (OR=2.19 95% Cl: 1.24-3.86, p=001).

Conclusion

These findings suggest that the prevalence of poor sleep quality and worsening mental health in Riyadh, Saudi Arabia was high during the COVID-19 lockdown, which articulates the requirement for raising the awareness, screening, and management of worsening sleep quality and mental health due to the unwholesome effect they may have on the individual’s health.

## Introduction

The COVID-19 pandemic became a worldwide burden of disease when the World Health Organization (WHO) regional office in China received notifications of pneumonia cases of unknown origin in December 2019 in Wuhan, China. In January 2020, the Chinese authorities announced that they have discovered the causative organism named severe acute respiratory syndrome coronavirus 2 (SARS-CoV-2) [1]. The gap in knowledge about the emerging disease caused dramatic changes in different countries around the world, and numerous measures were implemented by the government of Saudi Arabia to control the outbreak, including the ban on mass gatherings, the closure of the two holy mosques, and the transformation to online teaching and teleworking [2]. Riyadh city was going through many developmental changes prior to the pandemic in the socio-economic pattern, as different types of activities were set up which led to an increase in the number of citizens and tourists. Saudi Arabia relied on traditional public health measures to combat this pandemic of severe respiratory ailments. Quarantine has been one of the oldest and most efficient strategies among these measures for managing infectious disease pandemics; since it was effectively implemented in many other countries during the severe acute respiratory syndrome (SARS) pandemic in 2003 [3]. Workplace social distancing has been shown to shrink the overall number of influenza cases during previous influenza epidemics [4]. It was also found that school closure reduces influenza transmission and delays an epidemic peak [5]. As a result of the lockdown, many people experienced alterations in their daily routines, and lived in uncertainty, stress, and health insecurity during this eventful period [6]. The lockdown situation is likely to have a negative impact on the psychological behaviors and social experience of most people due to the requirement of physical and social distancing, job insecurity, and being separated from family and friends [7]. Moreover, this new life configuration led to significant consequences on sleep and mental health [8]. Sleep disturbances, including insomnia and overall poor sleep quality and quantity, result in detrimental effects on a person’s overall health [9,10]. It has also been proved that anxiety and depression can contribute to sleep disturbances or exacerbate sleep disturbances symptoms [11,12]. During the COVID-19 crisis, the psychological effect ranged from fear and uncertainty to involve a wide range of mental health disorders like insomnia, anxiety, and depression [13]. A previous study has reported mental health outcomes of healthcare providers during the COVID-19 pandemic in Saudi Arabia [14]. Looking forward, this study will evaluate the psychological effect and sleep disorders on participants living in Riyadh, Saudi Arabia, including healthcare providers. We aimed to assess the prevalence of sleep disturbance and the psychological impact associated with the spread of the COVID-19 in Riyadh, Saudi Arabia. 

## Materials and methods

A cross-sectional study was conducted in Riyadh, Saudi Arabia, to assess sleep disturbances and the psychological status during the COVID-19 pandemic. Data collection was carried out during the COVID-19 pandemic (between September 27th and October 10th, 2021); participants were chosen randomly and were asked to complete an Arabic online questionnaire survey that was published through social media platforms. The inclusion criteria was individuals above 18 years old living in Riyadh City and willing to participate in the study. Participants with psychotic and/or sleep disorders prior to the COVID-19 outbreak were excluded from the study. Data was collected and analyzed by Statistical Package for the Social Sciences (SPSS; IBM Inc., Armonk, New York). This study was approved by the institutional review board (IRB) Ethics Committee of King Fahad Medical City in Riyadh. All participants were provided informed online consent before answering the questions. 

Self-measurement

The structured questionnaire involved the following three sections: 1) demographics, 2) sleep quality and insomnia, and 3) anxiety and depression. The sociodemographic part involved the following characteristics: gender, age, marital status, occupation, comorbidities, smoking status, history of exposure to COVID-19, prior history of any psychological or sleep disorders, and COVID-19 vaccine status. Sleep quality and insomnia were measured by using two scales: Athens Insomnia Scale (AIS) and Insomnia Severity Index (ISI). AIS is a self-assessment psychometric instrument that was used to investigate sleep difficulties that the participants might have experienced during the COVID-19 pandemic. It involves eight statements that evaluate the assessment of full duration and quality of sleep. AIS also permits the estimation of stress caused by the experience of insomnia and the desire to sleep during the day and their interference with routine daily activities. A score of six or above indicates the presence of insomnia, while scores less than six indicate the absence of insomnia [15]. Insomnia Severity Index (ISI) is a seven-item self-report questionnaire assessing the nature, severity, and impact of insomnia. The items were rated between 0 (no problem) to four (very severe problem) by a five-point Likert scale, yielding a total score ranging from 0 to 28. A score less than eight indicates absence of insomnia and a score of eight or greater shows symptoms of insomnia [16]. The remaining section measured the psychological status of the participants by using the Hospital Anxiety and Depression Scale (HADS). The Hospital Anxiety and Depression Scale consisted of 14 items, which were divided into two sub-scales, HADS-A and HADS-D, measuring anxiety and depression, respectively. Each item was measured on a four-point scale, a score 0 of indicting "not present" and 3 indicating a "considerable event", yielding a total score ranging from 0 to 21 on each of these two sub-scales. A cutoff point of ≥8 was used. HADS Evaluates the symptom severity of anxiety and depression disorders. As evidenced; this scale can be applied to samples from the general population, general practice, and psychiatric patients [17].

Sample size

A sample size of 385 was needed for this cross-sectional study based on a response rate of 50%, a confidence interval of 95%, and a margin of error of 5%. The Cochrane formula was used to calculate sample size =$\sqsubset n= z^{2} \frac{\alpha }{2} \frac{SD^{2}}{d^{2}}\sqsupset$, where $Z=\frac{\alpha }{2}$= is standard normal variate (5% type 1 error), SD is standard deviation of variable, d is absolute error. 

Statistical analysis

SPSS version 25.0 software was used for data analysis. Categorical data was reported as frequency and percentages. Age of the participant, as well as AIS, ISI, Anxiety, and Depression scores, were presented as means and standard deviation (mean ± SD). The totals of AIS, ISI, Anxiety, and Depression scores were dichotomized according to the guidelines. Pearson’s correlation and linear regression predicted internal relationship of COVID-19 vaccine with the AIS, ISI and HADS characteristics. A Chi-square test was applied to measure the association between the sociodemographic characteristics and vaccination status with the underlying dependent variable, which were further loaded for binary logistic regression analysis. A p-value of ≤0.05 was considered statistically significant. The outcome was represented by an adjusted odds ratio, and all the inferences were drawn at 95% CI.

## Results

A total of 481 participants completed the survey, among which 82 (17.04%) were excluded due to data insufficiency or not fulfilling the inclusion criteria. The final sample thus consisted of 399 participants. The mean age was 34.70 ± 12.571 years, and predominant responses were from females (69.4%). The study sample was mostly made up of students (32.1%), and more than half of the participants (52.6%) were married. The majority of the participants (87%) had two doses of the COVID-19 vaccine, and 310 (77.7%) were not previously infected by COVID-19. Detailed socio-demographic information is presented in Table 1.

**Table 1:** Characteristics of the respondents (total sample=399)

Characteristic	Description	n (%)
Gender	Male	122 (30.6)
	Female	277 (69.4)
Marital status	Never married	170 (42.6)
	Married	210 (52.6)
	Widowed	8 (2.0)
	Divorced	11 (2.8)
Occupation	Student	128 (32.1)
	Government sector	87 (21.8)
	Privates sector	26 (6.5)
	Healthcare	5 (1.3)
	Freelance	7 (1.8)
	Retired	44 (11.0)
	Unemployed	102 (25.6)
Age (years)	<35	199 (49.9)
	≥35	200 (50.1)
	min - max	19 - 63
	mean ± SD	34.7 ± 12.6
	median (p25 - p75)	35 (22 - 45)
Previously infected by COVID-19	Yes	89 (22.3)
	No	310 (77.7)
COVID-19 vaccine	1 dose	39 (9.8)
	2 doses	347 (87.0)
	No	13 (3.3)
Smoking	Yes	49 (12.3)
	No	350 (87.7)

**Figure 1:**
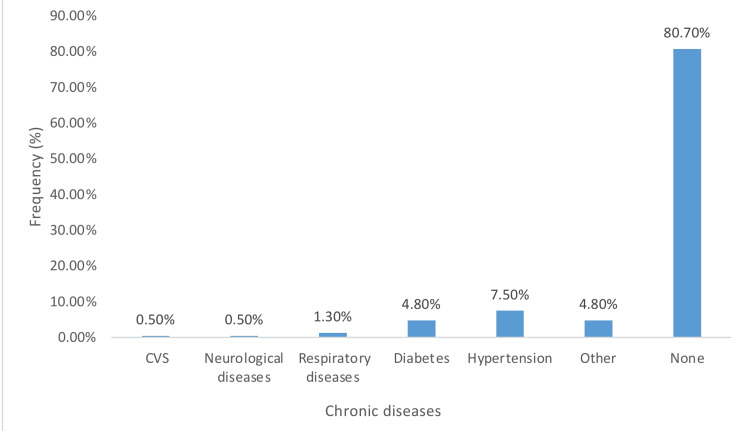
Frequency of chronic diseases CVS: cardiovascular system

As shown in Figure 1,** **a** **total of 322 participants (80.7%) were free from chronic diseases, 7.5% reported having hypertension, and 4.8% had diabetes. The prevalence of anxiety, depression, and insomnia by the AIS and ISI scales in Riyadh residents during the COVID-19 pandemic was 38.6% (n=154), 33.2% (n=132), and 54.9% (n=219), and 64.2% (n=256) respectively, as depicted in Table 2.

**Table 2:** Total scoring of anxiety, depression, and insomnia among the total sample n=399 HADS: Hospital Anxiety and Depression Scale, HADS-A: anxiety, HADS-D: depression, AIS: Athens Insomnia Scale, ISI: Insomnia Severity Index

Characteristic	Description	n (%)
HADS-A normal	<8	245 (61.4)
HADS-A abnormal	≥8	154 (38.6)
HADS-D normal	<8	267 (66.9)
HADS-D abnormal	≥8	132 (33.1)
AIS normal	<6	180 (45.1)
AIS abnormal	≥6	219 (54.9)
ISI normal	<8	143 (35.8)
ISI abnormal	≥8	256 (64.2)

The mean HADS score for anxiety was 1.91 ± 0.51. A higher anxiety score (76.7%) was reported with item two (feeling restless and always have to be on the move), with a mean score of 1.13 ± .91. The mean HADS score for depression was 2.57 ± 0.89. The highest depressive symptom (87.2%) was reported with item one (the feeling of being slowed down), with a mean score of 1.24 ± .76. The overall mean for AIS was .84 ± .52; most participants (79.4%) suffered from insomnia due to higher scores with item one (sleep induction), with a score of 1.17 ± .83. The overall mean for ISI was 1.38 ± .76, and the highest score (67.9%) was reported with item four (How satisfied/dissatisfied are you with your current sleep pattern?), with a mean score of 2 ± 1.12. 

Table 3 illustrates the association of socio-demographics with anxiety, depression, and insomnia. A logistic regression model revealed higher anxiety among females (OR=1.79, 95% Cl:0.98-3.27, p=0.641), having chronic diseases (OR=1.73, 95% Cl:0.97-3.06, p=0.39), and participants who weren’t previously infected by COVID-19 were more likely to have anxiety than previously infected group (OR=1.7, 95% Cl: 1.04-2.8, p=0.033). In relation to other occupations, insignificantly higher anxiety symptoms were noticed in students (46.9%) than in the working-class (36%, p=0.08); however, the retired group had lesser levels of anxiety (OR=0.31, 95% Cl: 0.11-0.89, p=0.08). Anxiety levels were higher among married than single participants (OR=1.18, 95% CI: 0.52-2.66, p=0.34). Depressive symptoms in association with gender were more likely among females than males (OR=1.24, 95% Cl:0.66-2.33, p=0.43). Moreover, participants with comorbidities were significantly at higher risk to have symptoms of depression in comparison to subjects free from chronic diseases (OR=2.19 95% Cl: 1.24-3.86, p=0.01). Depression was less likely among the age of 35 years old and above (OR=0.83, 95% Cl:0.4 -1.74, p=0.54) and the retired group (OR=0.34, 95% Cl:0.11-1.02, p=0.68).

**Table 3:** Prevalence of anxiety, depression, and insomnia during COVID-19 pandemic, and the results of univariate and multivariate logistic regression analysis OR: odds ratio; 95% CI: 95% confidence interval; AIS: Athens Insomnia Scale, ISI: Insomnia Severity Index; *p<0.05; **p<0.001; a: widowed, divorced

Characteristic	Description	AIS	ISI	Anxiety	Depression
		OR 95% CI	OR 95% CI	OR 95% CI	OR 95% CI
Gender	Male	-	-	-	-
	Female	1.92 (1.06 - 3.5)*	1.4 (0.76 - 2.58)	1.79 (0.98 - 3.27)	1.24 (0.66 - 2.33)
Age (years)	< 35	-	-	-	-
	≥ 35	0.65 (0.32 - 1.33)	0.6 (0.29 - 1.27)	0.72 (0.35 - 1.47)	0.83 (0.4 - 1.74)
Marital status	Never married	-	-	-	-
	Married	1.03 (0.47 - 2.27)	0.88 (0.39 - 1.99)	1.18 (0.52 - 2.66)	1.82 (0.77 - 4.3)
	Other ^a^	3.32 (0.88 - 12.57)	0.92 (0.26 - 3.25)	1.79 (0.51 - 6.34)	2.77 (0.77 - 9.99)
Occupation	Student	-	-	-	-
	Working-class	0.58 (0.28 - 1.2)	0.84 (0.39 - 1.8)	0.56 (0.27 - 1.19)	0.53 (0.24 - 1.19)
	Retired	0.18 (0.06 - 0.51)**	0.65 (0.23 - 1.81)	0.31 (0.11 - 0.89)*	0.34 (0.11 - 1.02)
	Unemployed	0.46 (0.21 - 1.01)	1.14 (0.49 - 2.63)	0.56 (0.25 - 1.26)	0.72 (0.3 - 1.69)
Comorbidities	No	-	-	-	-
	Yes	1.53 (0.86 - 2.74)	1.69 (0.93 - 3.07)	1.73 (0.97 - 3.06)	2.19 (1.24 - 3.86)*
Previously infected by COVID-19	Yes	-	-	-	-
	No	1.12 (0.67 - 1.86)	1.74 (1.01 - 3.02)*	1.7 (1.04 - 2.8)	1.11 (0.66 - 1.86)
Vaccine status	No	-	-	-	-
	Yes	4.86 (1.26 - 18.67)*	8.29 (2.15 - 31.94)*	2.49 (0.65 - 9.52)	2.01 (0.52 - 7.84)
Smoker	Yes	-	-	-	-
	No	1.74 (0.83 - 3.64)	1.53 (0.71 - 3.3)	2.1 (1 - 4.41)	1.65 (0.77 - 3.52)

As shown in Table 3, AIS-insomnia symptoms among students were significantly higher (68%) than other occupations; working-class 53.6%, retired 34.1%, and unemployed 49.0% (p<0.001). Insomnia was significantly higher among COVID-19 vaccinated group than in the non-vaccinated group (OR=4.86, 95% CI: 1.26-18.67, p=0.019), single participants had higher insomnia (62.9%) than married participants (47.1%, p=0.004). However, insomnia was significantly less likely in the retired (OR=0.18, 95% Cl: 0.06-0.51, p<0.001). Insomnia was higher among students (68%) than among the working class (53.6%, p=<0.001). Furthermore, insomnia was less likely among the age of 35 years old and above (OR=0.65, 95% Cl: 0.32-1.33, p=0.003). According to ISI, insomnia was significantly more likely among the COVID-19 vaccinated (OR=8.29, 95% CI: 2.15-31.94, p=0.002), people not previously affected with COVID-19 (OR=1.74, 95% CI: 1.01-3.02, p=0.084), females (OR=1.4, 95% Cl: 0.76-2.58, p=0.87), and the unemployed (OR=1.14,95% Cl:0.49- 2.63, P=0.14). Insomnia was significantly higher among participants aged <35 years old (69.3%) than in those aged 35 years old and above (59%, p=0.031).

**Figure 2:**
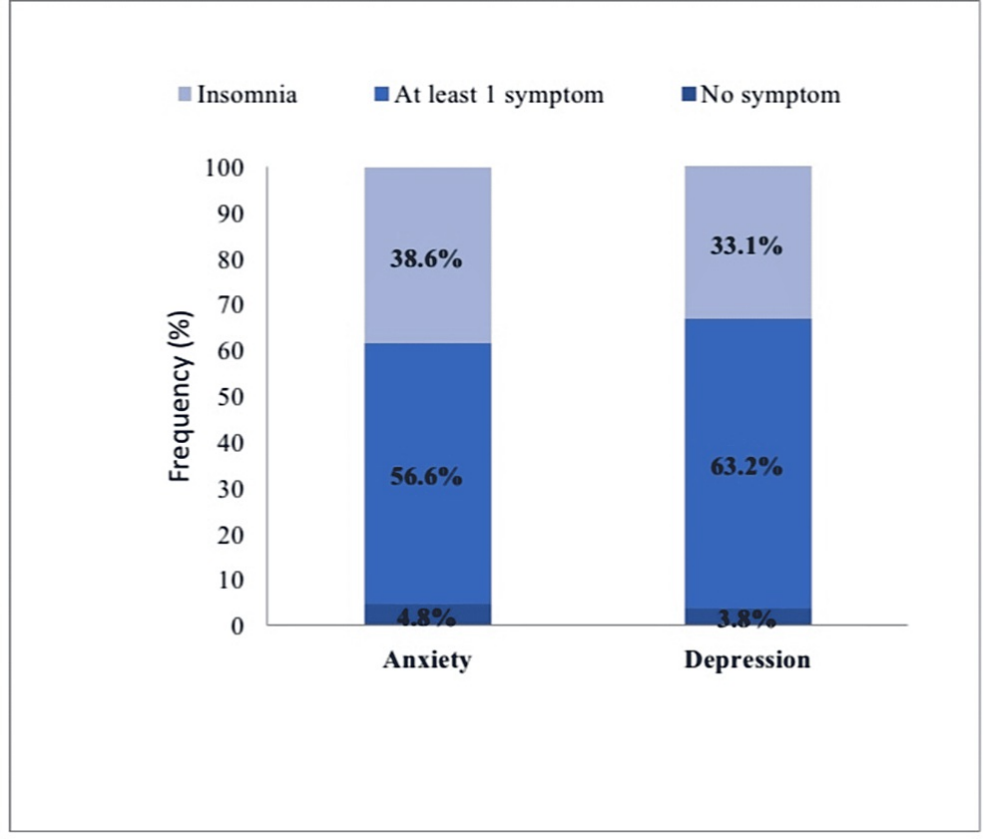
Prevalence of sleep disturbance among participants with anxiety and depression

The prevalence of sleep disturbances among participants with anxiety and depression during the COVID-19 pandemic is shown in Figure 2. 

Correlation analysis

**Figure 3:**
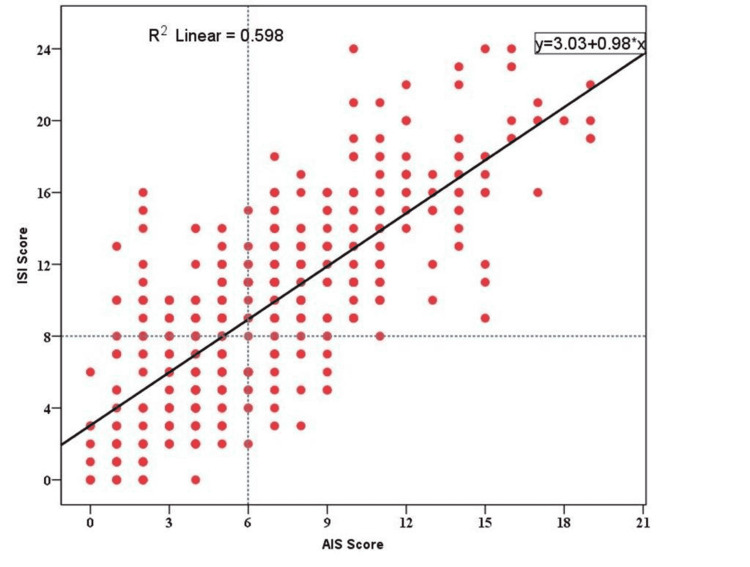
Relationship between ISI scores and AIS scores in the anxiety group AIS: Athens Insomnia Scale, ISI: Insomnia Severity Index

The significant positive correlation between AIS and ISI (r=0.76, p<0.001) indicates their high level of interdependence, as shown in Figure 3.** **Of the participants, 48.1% had insomnia, which had a major mapping within the score of AIS ≥ 6 and ISI ≥ 8. Moreover, a significant positive correlation was observed between poor sleep quality and anxiety (r=0.56, p<0.001), as well as anxiety and depression (r=0.69, p<0.001).

Table 4 shows a non-significant increase in insomnia among participants who were vaccinated with one dose (76.9%) in relation to participants who received two doses (64.3%, p=0.11) Anxiety nor depression bore any significant relation with vaccine doses. Vaccination with one dose or two doses had a significant positive correlation (r=0.47, p<0.001). Furthermore, participants vaccinated with one dose had a significant negative correlation with insomnia (r=0.157, p=0.002). On the other hand, participants vaccinated with two doses had insignificant negative correlations with anxiety (r= -.014, p=0.77) and depression (r= -.044, p=0.37). 

**Table 4:** Correlation analysis regarding the impact of COVID-19 vaccine HADS: Hospital Anxiety and Depression Scale, HADS-A: anxiety, HADS-D: depression, AIS: Athens Insomnia Scale, ISI: Insomnia Severity Index *p<0.05

	Single dose (n=39)		Two doses (n=347)	
	n(%)	r	n(%)	r
AIS ≥6	22 (56.4)	.117*	194 (55.9)	0.053
ISI ≥8	30 (76.9)	.157*	223 (64.3)	0.006
HADS A ≥8	18 (46.2)	0.059	133 (38.3)	-0.014
HADS D ≥8	17 (43.6)	0.039	112 (32.3)	-0.044
Two doses	-	.474*	-	-

## Discussion

The present study evaluates the impact of the COVID-19 lockdown on mental health and sleep disturbances in Riyadh. Mental burden was higher among females, students, participants with chronic diseases, COVID-19 vaccinated, and in those not previously infected by COVID-19. On the other hand, fewer symptoms were reported in the retired group and among participants aged 35 years old and above. Moreover, students were more likely to have insomnia compared to other occupations, and the retired group was 66% less likely to have depressive symptoms.

Alharbi et al. assessed the prevalence of insomnia at 54.4% among the residents of Saudi Arabia by using the AIS scale, which was very much consistent with our findings which showed that 54.9% of residents had insomnia during the pandemic. Moreover, they found that single participants had insomnia more than married participants, which was in accordance with our results where single participants had higher AIS scores (p=.004) [2].

In Greece, Voitsidis et al. declared that 37.6% had insomnia which was less than our findings of 54.9%. Similar to our findings, with respect to the sociodemographic factors, Voitsidis et al. found that women had significantly higher insomnia scores than men (p=0.001) and claimed that women were more vulnerable at times of stress. Moreover, they observed significantly higher insomnia levels among adults below the age of 30 years old [18]. Additionally, they found that residents living in urban areas had a higher prevalence of insomnia than rural citizens, which was confirmed by Idrissi et al., who stated that insomnia prevalence can be significantly predicted according to the geographical location of the participants observed in Morocco [18,19]. Idrissi et al. also linked insomnia with chronically diseased participants. They also reported that gender and age constitute significant predictors of depressive symptoms; females showed higher depressive symptom scores (OR=0.53, 95% Cl: 0.40-0.71, p<0.05), and so did those under the age of 35 (OR=0.42, 95% Cl: 0.32-0.57, p<0.05), which is closely related to our results [19].

The impact of COVID-19 on sleep quality was observed to be higher among females and students. This is in agreement with a study that was conducted recently in Italy that showed a higher impact on both females and students, therefore recommending psychological support [20]. Our study revealed a high prevalence of anxiety (38.6%) and depression (33.1%). However, we could not identify the associations linked to this high prevalence. Our findings in terms of anxiety prevalence were somehow consistent with those found in the literature [21-23]. Being retired and not having virtual work during the pandemic showed 37% less depressive symptoms. Recent systematic reviews show that healthcare professionals and women are at increased risk of depression, anxiety, poor sleep quality, and psychological distress [21]. In Turkey, Özdin et al. reported a high prevalence of anxiety (45.1%) and depression (23.6%) among the Turkish population; while depression in our finding was higher, we believe that because he used a higher cutoff point of 10 while in this research eight was used as a cutoff point which led to underestimation in the prevalence of depression. He also noted that anxiety and depression scores were significantly higher among women, a finding which is consistent with that of White et al., who studied the prevalence of anxiety among the people of the United Kingdom [22,23]. Our results also showed that females were more likely to have anxiety and depression. In Poland, Chodkiewicz et al. reported a slightly lower prevalence of anxiety and depression (32.69% and 23.14%, respectively). They assessed anxiety and depression using the same questionnaire used in our study (HADS). Moreover, they used the same cutoff point in our study (≥8) for depression, and a higher cutoff point of (≥11) for anxiety, compared to the one used in our study (≥8), which might have led to this modest underestimation in the prevalence of anxiety [24]. 

Limitations

The current study has some limitations. First, due to the cross-sectional nature of the research, a clear perspective on the changes and variations in the respondents’ mental health and sleep quality for the long-term effects of the lockdown was difficult to estimate. Hence, further prospective studies are needed to assess the long-term consequences of the pandemic in a longitudinal study design. Secondly, the study was conducted using an online questionnaire survey, which could have introduced some sort of selection bias since the individuals with no internet access or who did not use smartphones were unable to participate. When compared to an interview, the survey relied on self-report, which might have introduced systematic bias and yielded different results.

## Conclusions

Some of the consequences of the lockdown due to the COVID-19 pandemic were associated with worsening sleep quality and mental health. Medical attention is required to deal with COVID-19 lockdown’s impact on sleep quality and mental health because they are necessary for the quality of life and productivity. This study showed a high prevalence of poor sleep quality and worsening mental health during the COVID-19 lockdown, which articulates the requirement of raising the awareness, screening, and management of worsening sleep quality and mental health due to the unwholesome effect they may have on the individual health.
